# Accelerated institutionalization of an adolescent sexual and reproductive health (ASRH) intervention in Tanzania: Findings from a mixed-methods evaluation

**DOI:** 10.3389/fgwh.2023.942418

**Published:** 2023-03-15

**Authors:** Meghan Cutherell, Juliana Bwire, Edwin Mtei, Abednego Musau, Catherine Kahabuka, Isabellah Luhanga, Augustino Julius, Gerald Kihwele

**Affiliations:** ^1^ Population Services International (PSI), Washington, DC, United States; ^2^ Population Services International (PSI) Tanzania, Dar es Salaam, Tanzania; ^3^ Itad, London, United Kingdom; ^4^ CSK Research Solutions, Dar es Salaam, Tanzania; ^5^ Tanzania Ministry of Health (MOH), Dodoma, Tanzania

**Keywords:** adolescent sexual and reproductive health (ARSH), institutionalization, Tanzania, sustainability, scale-up, vertical integration

## Abstract

**Introduction:**

From 2018 to 2020, Adolescents 360 (A360), aiming to increase demand for and voluntary uptake of modern contraception among adolescent girls 15–19 years, designed and scaled an intervention in Tanzania (Kuwa Mjanja) to 13 regions through project-funded expansion. In 2020, the project began to develop a strategy for its follow-on phase, focusing on program sustainability. In this process, funder priorities led to a decision to exit A360's programming in Tanzania over a 15-month exit period. A360 elected to pursue a process of expedited institutionalization of Kuwa Mjanja into government systems during this period.

**Materials and methods:**

The institutionalization process was facilitated in 17 local government authorities in Tanzania. Quantitative and qualitative data were gathered and analyzed including time-trend analysis of routine performance data, statistical analysis of two rounds of client exit interviews, and thematic analysis of qualitative research.

**Results:**

The sociodemographic characteristics of adolescent girls reached under government-led implementation were comparable to those reached by A360-led implementation. Intervention productivity decreased under government-led implementation but remained consistent. Adopter method mix shifted slightly toward greater long-acting and reversible contraceptive uptake under a government-led model. Factors that enabled successful institutionalization of Kuwa Mjanja included the presence of youth-supportive policies, the establishment of school clubs which provided sexual and reproductive health education, commitment of government stakeholders, and appreciation of adolescent pregnancy as a problem. Some intervention components were important for program effectiveness but proved difficult to institutionalize, primarily because of resource constraints. Lack of adolescent sexual and reproductive health (ASRH)-focused targets and indicators disincentivized Kuwa Mjanja implementation.

**Discussion:**

There is significant potential in operationalizing user-centered ASRH models within government structures, even in a narrow time frame. A360 saw similar performance under government-led implementation and fidelity to the unique experience that the program was designed to deliver for adolescent girls. However, beginning this process earlier presents greater opportunities, as some aspects of the institutionalization process that are critical to sustained impact, for example, shifting government policy and measurement and mobilizing government resources, require heavy coordination and long-term efforts. Programs pursuing institutionalization in a shorter time frame would benefit from setting realistic expectations. This may include prioritizing a smaller subset of program components that have the greatest impact.

## Introduction

Global health programs that aim to improve adolescent sexual and reproductive health (ASRH) have generated important learning over the past three decades (1). Still, questions remain about how to best design, implement, and scale sustainable programming that demonstrably responds to the needs and experiences of adolescents (2). Scaling up of effective adolescent-focused programming can take multiple forms, with the most effective scale-up combining vertical and horizontal scale-up approaches (3). Examples of vertical scale-up of adolescent-focused programming through institutionalization, or adoption through political, policy, budgetary, regulatory, and other health system changes, exist (4–10), but documentation of scale-up processes tend to focus on horizontal scale-up through expansion or replication rather than on the process and milestones for institutionalization (2). What is known is that the time horizon for effective institutionalization is generally long—some cite a 17-year research to practice gap (11), and others reference an institutionalization time horizon of 10–15 years (12)—and that embedding evidence-based programming into government systems is a complex endeavor (13, 14).

**Figure 1:**
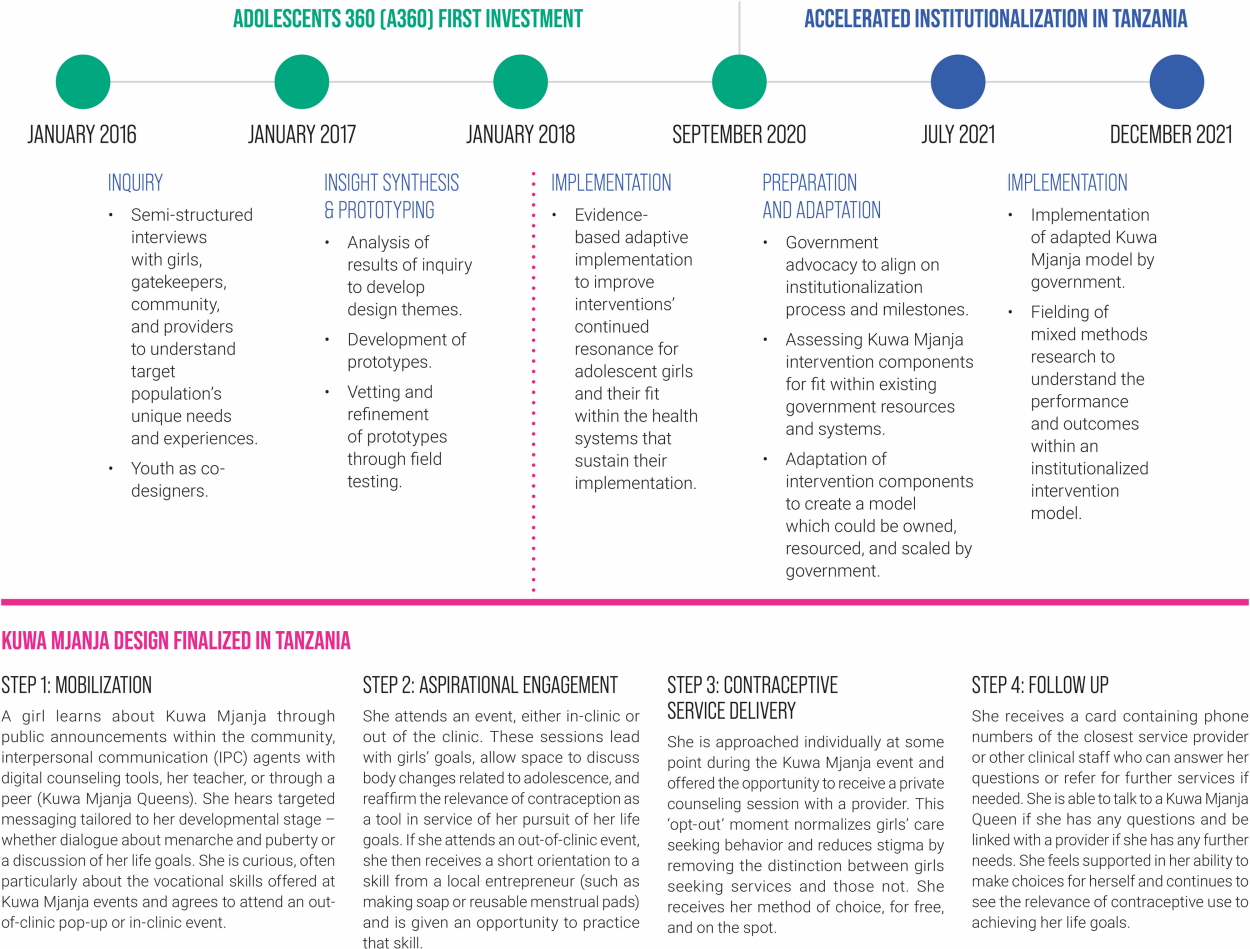
A360 Tanzania timeline and Kuwa Mjanja implementation approach.

From January 2016 to September 2020, Population Services International (PSI) implemented the Adolescents 360 (A360) project, which worked directly with youth to design and deliver interventions that increase demand for, and voluntary uptake of, modern contraception among adolescent girls aged 15–19 in Ethiopia, Nigeria, and Tanzania. During A360's implementation period, the project's interventions were scaled up through a process of expansion into new geographic areas. In this expansion, A360 partnered extensively with government and leveraged existing government structures and resources, for example, using only public sector providers for service delivery and conducting data and service quality audits and supportive supervision in partnership with government. However, scale-up was still driven primarily by project resources. In Tanzania, A360 designed Kuwa Mjanja, “Be Smart” in Swahili, through a multidisciplinary human-centered design (HCD) process (Figure 1). Throughout the course of the initial A360 investment, Kuwa Mjanja was scaled across 13 regions in Tanzania and reached over 300,000 adolescent girls with SRH services, with over 220,000 of these girls voluntarily adopting a modern method of contraception.

In early 2020, A360 undertook a robust co-creation process with its funders to design a follow-on project investment that would focus on institutionalization of its interventions into government systems and further scale-up into new sites through a process of government-led expansion, with the goal of pursuing sustainability. Early in this co-creation process, A360 partnered with ExpandNet to refine its thinking and approaches in preparation for the pursuit of institutionalization, with Tanzania included as part of this planning and co-creation process. However, a shift in the geographic priorities of the project's funders midway through this co-creation process led to a decision for A360 to exit its programming in Tanzania. The project was given 12 months after the end of the first investment for this exit period, along with an additional three months for close-out. Leveraging the planning completed earlier in the co-creation process and capitalizing on existing government buy-in, A360 elected to pursue a process of expedited institutionalization of Kuwa Mjanja into government systems during this exit period. Additional scale through government-led expansion was not considered given the limited time. The key learning question for this period was: What successes can be achieved and what tradeoffs must be made to pursue an accelerated process of institutionalization of Kuwa Mjanja into government systems over the course of a single year? Through a participatory process with government, A360 mapped out a process for how to pursue institutionalization. A360 also crafted a learning agenda to contribute to the global evidence base around the process and milestones for institutionalization. This publication details the process and results of this accelerated institutionalization period.

## Materials and methods

### Study setting

This institutionalization process and accompanying evaluation took place across in 17 local government authorities (LGAs) in Tanzania, including Kalambo, Nkasi, Sumbawanga, and Sumbawanga Urban in Rukwa; Mlele, Mpanda Urban, Mpimbwe DC, Nsimbo, and Tanganyika in Katavi; and Madaba, Mbinga TC, Mbinga DC, Matumbo, Nyasa, Songea, Songea urban, and Tunduru in Ruvuma. These locations were selected due to multiple factors including high rates of teenage pregnancy, Ministry of Health (MOH) prioritization, and limited saturation of other implementing partners. A360 was implemented in all 17 LGAs from 2018 to 2020 during the implementation phase of its first investment.

### Institutionalization process

Drawing from the current evidence base around institutionalization, A360 recognized that its processes needed to be phased and structured (13). A biphasic approach consisting of adaptation and implementation phases was employed. The first phase (adaptation) was designed to facilitate a modification of the existing Kuwa Mjanja intervention for better fit with government systems and constraints. A full description of the intervention, its constituent components, and the girls’ user journey prior to the intervention's adaptation are described elsewhere (15). The second phase (implementation) was designed to provide a structured period for evaluating the results of institutionalization efforts.

#### Adaptation (Q1–Q2 2021)

**Figure 2:**
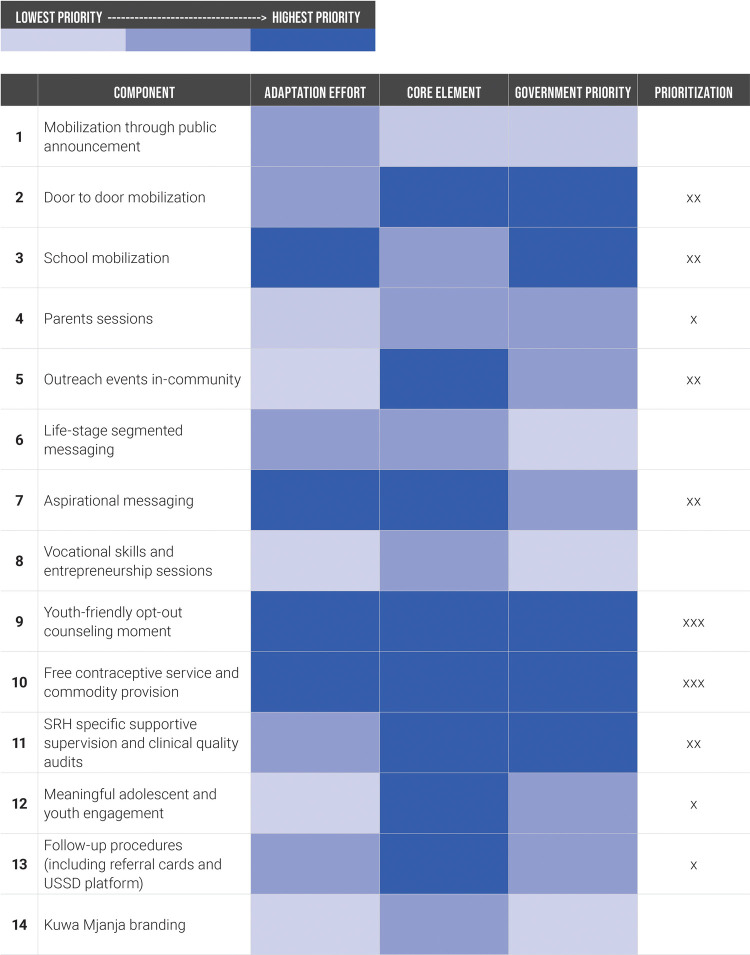
Kuwa Mjanja component prioritization during the institutionalization adaptation phase.

Between January and June 2021, A360 pursued intensive adaptation to the Kuwa Mjanja model to improve fit within existing government systems and resources. In collaborative working sessions, A360 and government stakeholders separated the intervention into its constituent parts and used a consensus building process to assess each component using three criteria. This included (1) how easy it would be to adapt the component to be operationalized within existing government resources, (2) how “core” or important the component was to program effectiveness, and (3) how aligned the component was with existing government priorities and activities (Figure 2). The components that were scored highest priority in at least one category were taken into the adaptation process, though the intensity of focus was greater for those components which were high priority in multiple categories.

A360 and government counterparts then worked to map out how each of the prioritized components would be operationalized through existing government resources and systems. The ideas for operationalizing these components were tested throughout this adaptation period with the government and A360 continuing to identify and make iterations throughout. The District Reproductive and Child Health Coordinator (DRCHCo) for each district convened monthly meetings involving other government counterparts involved in Kuwa Mjanja implementation as well as A360 staff to discuss the results of the testing and adaptation process and identify continued iterations that could be made. A360 continued to monetarily support some aspects of program implementation while adaptations were tested and finalized. For example, when the government identified an opportunity to use community health workers (CHWs) to take up mobilization for Kuwa Mjanja, they requested that A360 support sensitization of CHWs on mobilization of adolescent girls and provide incentives for them for playing this role until alternative methods of compensation could be identified and mobilized. Often, government stakeholders met independent of A360 to monitor the adaptation progress and identify opportunities for iteration. During this phase, A360 was still present at most Kuwa Mjanja events, observing whether adaptations were successfully implemented, gathering data on user experience with these adaptations, and providing mentorship and capacity building to government staff on Kuwa Mjanja implementation.

A360 captured the adaptations that were tested and opportunities for further learning through adaptation audits, which were conducted twice during this phase. After each adaptation audit, A360 and government stakeholders jointly made decisions about whether to continue pursuing adaptation to each intervention component. Most components continued to be adapted until the end of the adaptation phase. The final government-led model (Table 1) was taken into the next phase of the institutionalization process. “A360-led model” refers to the Kuwa Mjanja design and operating model, which was implemented under the initial A360 investment phase (15). Though this model leveraged on existing government structures, its implementation was still primarily led by and funded through A360. In contrast, “government-led model” refers to a model that was directed by and funded by government with A360 having transitioned into a purely technical assistance and capacity building role.

**Table 1:** Government-led Kuwa Mjanja user journey compared to A360-led user journey.

User journey stage and description	A360-led Kuwa Mjanja model	Government-led Kuwa Mjanja model (most significant adaptations are shown in bold)
Mobilization “*I’m intrigued”* A girl hears about A360's programming and feels it is relevant. She feels engaged by the support A360 provides to work toward her goals for her life. She feels supported by her influencers to attend A360 programming.	• Girls hear about Kuwa Mjanja through public announcement within the community or A360 mobilizers with digital counseling tools. • Girls engage with adolescent co-implementers, Kuwa Mjanja Queens, who have been recruited from prior program users. • Girls may also hear about Kuwa Mjanja from a teacher who informs her about events and the opportunity to participate. • A360 engages girls’ key influencers (primarily parents) with sessions to orient them to Kuwa Mjanja, build empathy for adolescent girls’ experiences, and prompt support for their contraceptive use.	• **Most girls hear about Kuwa Mjanja through a CHW,** a service provider or a teacher. • Clubs established in schools and run by teachers trained to provide youth-friendly ASRH education provide avenues for referral to Kuwa Mjanja events. • CHWs walk door to door to invite girls to events. Girls often invite their friends after being invited by CHWs. • **Key influencer sessions are no longer implemented because there are no existing government structures which can sustain them.**
Aspirational engagement “*I’m inspired and motivated”* She feels that she has a safe and supportive space to plan for her life goals. She understands how contraception can be a tool to help her achieve these goals.	• Girls come to a Kuwa Mjanja event either in a clinic or in the community and are introduced to the “nanasi” story (meaning pineapple in Swahili), demonstrating how girls can “stand tall, wear their crown, and be a role model.” • Sessions lead with girls’ goals and reaffirm the relevance of contraception in pursuit of life goals. • If a girl attends an out-of-clinic event, she receives an orientation to a skill from a local entrepreneur (such as making soap or reusable menstrual pads) and is provided with an opportunity to practice that skill.	• **All events are now in public sector clinics.** After coming to the Kuwa Mjanja event, girls learn about different SRH-related topics from trained youth-friendly health providers. • The experience is de-branded, though messaging elements from the original Kuwa Mjanja brand remain. • Girls understand from health providers how contraception can help them plan for a better future family and reach their goals. • Some girls also receive skills building during events, though not **all events include vocational and entrepreneurial skills demonstrations**.
Contraceptive counseling and service delivery “*I feel respected and safe”* She is invited to participate in a contraceptive counseling session. She feels that her self-defined goals are respected by the provider. The provider messages contraceptive methods in order of her interest and emphasizes their key benefits.	• Each girl is approached individually during the event and given an opt-out private counseling session with a provider. • The opt-out session provides all girls with an opportunity to talk with a provider and works to de-stigmatize SRH care-seeking behaviors. • If desired, she receives her contraceptive method of choice, for free, on the spot.	• During Kuwa Mjanja events, all girls receive an opt-out private moment with a youth-friendly provider to receive contraceptive counseling. • Wherever feasible, girls are provided with the ability to choose from a full range of contraceptive methods, though stock outs of certain commodities limit the basket of contraceptive methods available in some cases.
Follow-up “*I feel supported”* She feels able to come back to receive follow-up services whenever she has questions or needs more contraception. She continues to see contraception as relevant to achieving her life goals.	• A girl receives a card containing phone numbers of the closest service provider or clinical staff who can answer her questions or refer her for services. • She has the ability to talk to a Kuwa Mjanja Queen if she has any questions, who can connect her with a provider if she has further needs.	• Providers utilize standard government follow-up procedures including providing girls with dates for revisits depending on method uptake. • Limited quantities of referral cards are available for use at government facilities to remind girls when it is time to return for follow-up services. • **Many options for follow-up are removed.**

A360, Adolescents 360; CHW, community health worker; ASRH, adolescent sexual and reproductive health; SRH, sexual and reproductive health.

#### Implementation (Q3–Q4 2021)

The government-led Kuwa Mjanja model finalized during the adaptation phase was taken into an implementation phase from July to November of 2021. Though some sites began to implement government-led Kuwa Mjanja events during the adaptation phase, A360-led events did not phase out until July. During this phase, A360 stepped back from a direct implementation role, ceasing any A360-led Kuwa Mjanja event implementation and observing what happened when the government was solely responsible for implementation. Planning processes were led by the DRCHCos, who developed monthly plans for the frequency and location of Kuwa Mjanja events within their catchment area. An A360 staff member attended most events to provide suggestions to service providers and other facility staff that could improve implementation. At the request of district and regional governments, A360 staff also conducted on-the-job training in youth-friendly and long-acting and reversible contraceptive (LARC) service provision and conducted abbreviated quality audits while at the facility, taking note of any potential concerns regarding the quality of services. Direction was given to A360 staff to provide advice and technical assistance to government but not to directly implement any program components. The one exception to this was the pairing of Kuwa Mjanja Queens with CHWs in some districts to continue capacity building in mobilization of adolescent girls. Kuwa Mjanja Queens were adolescent girls (15–19 years old) with existing social networks trained by A360 to support the mobilization of their peers and implementation of the out-of-clinic events in the geographies they reside. Though PSI stopped engaging Kuwa Mjanja Queens directly around 1–2  months into this phase, the government in some areas continued to invite or engage this cadre directly. This engagement, though promising, did not include continued payment for this cadre and did not continue after the end of the implementation phase. During this phase, responsibility for data collection was shifted from A360 to the health facility staff. A360 used government DHIS2 forms routinely to collect data before this accelerated institutionalization period, and also played a heavy role in data collection and data quality checks. The project continued use of government DHIS forms within this implementation phase, but data collection and data quality responsibilities shifted to government.

### Data collection methods

Both quantitative and qualitative data were gathered during the institutionalization process. Routine performance data, including aggregate contraceptive uptake among adolescent girls aged 15–19 (program adopters) as well as a disaggregation of method mix of adopters, were collected across all 17 LGAs through government health management information systems (HMIS). This included 54 health facilities in Rukwa, 63 in Katavi, and 94 in Ruvuma. For data unavailable from HMIS, including program attendance and individual age disaggregation of adopters, A360 utilized a tablet-based data collection system called “Connecting with Sara.” These data points were complementary and not routinely collected at every Kuwa Mjanja activity.

A360 conducted two rounds of structured client exit interviews (CEIs). The first surveyed attendees at A360-led Kuwa Mjanja activities in January 2021 (*n* = 413) across eight districts in four regions (Mwanza, Rukwa, Ruvuma, and Katavi). The second surveyed attendees at government-led activities in November 2021 (*n* = 317) across eight districts in three regions (Rukwa, Ruvuma, and Katavi). CEI participants were adolescent girls aged 15–19 years who were sampled consecutively by trained female enumerators immediately after they exited from selected service delivery points (health facilities) where contraceptive counseling and methods were provided through the Kuwa Mjanja intervention. Additionally, qualitative data were collected in October and November 2021 through key informant interviews (KIIs) with purposely selected and consented government officials (*n* = 36) and PSI staff members (*n* = 18) and focus group discussions (FGDs) involving adolescent girls (*n* = 17). Government stakeholders were individuals who had been working closely with the project team, were approached by the study team, and consented individually. Adolescent girls interviewed were located in the study regions, had participated in or were served through project activities, and were recruited through health providers. KIIs and FGDs were moderated by trained female qualitative researchers. PSI staff who were interviewed were either based in one of these regions or in the PSI Tanzania main office in Dar es Salaam. The learning aims of this research were to understand (1) the barriers and enablers to institutionalization of Kuwa Mjanja within government systems and (2) what changes needed to be made to the model and A360's ways of working to pursue institutionalization.

**Figure 3:**
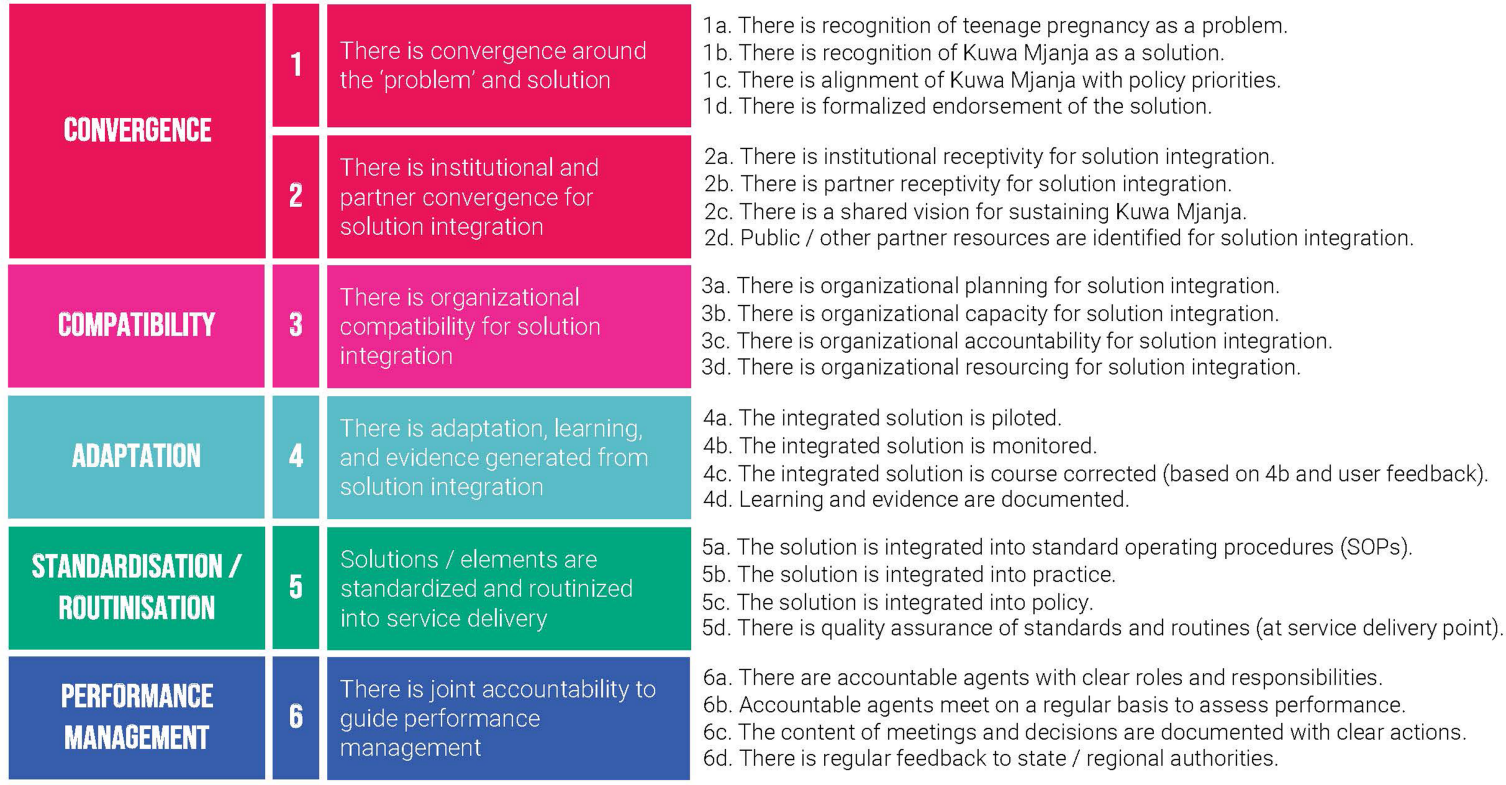
A360 institutionalization milestone framework.

To monitor progress throughout, A360 utilized an institutionalization milestone framework (Figure 3), which was developed in 2019 through a literature review of existing definitions and pathways for sustainability, drawing heavily from existing tools developed by ExpandNet (16, 17). A360 used a consensus building process with project and government stakeholders to assess institutionalization progress using this framework on a quarterly basis from January to September 2021. In Q4 2021 during qualitative data collection, key informant interviews with government stakeholders were used to develop an external set of scores using this framework.

### Measures

The CEI survey was interviewer-administered and contained questions to obtain data on client demographics (such as age, socioeconomic status, marital status, parity, and education) and client experience with the intervention, including quality of service delivery. Facilitation guides employed to moderate the KIIs covered themes related to implementation experience including the adaptation and implementation process, facilitators, and missed opportunities for institutionalization. Guides for FGDs were fielded to eligible adolescent girls aged 15–19 and were focused on their experiences as they navigated throughout the various touch points in the Kuwa Mjanja user journey. The milestone framework includes six domains—problem and solution convergence, institutional convergence, compatibility, adaptation, standardization/routinization, and performance management. Each domain included a set of four indicators scored on a scale from 0 to 3 as illustrated in Figure 3. Performance data for both models were collected routinely by providers using MOH family planning registers and aggregated monthly into summary reporting tools uploaded to the national HMIS database. Data included number of adopters, their ages, the methods they received, and whether repeat or new adopters. Though data on method switching and continuation are also critical to understand whether girls are being supported to use contraception throughout the period they are in need, these data points were not available within routine HMIS. Basic demographic details of adolescent girls attending program events was captured using an attendance register built on PSI-owned electronic devices and maintained by mobilizers. All data were de-identified before they were analyzed.

### Data analysis

Routine performance data generated were analyzed in excel and presented as dashboards on District Health Information System 2 (DHIS2), utilizing primarily time-trend analysis. CEI data were entered into SurveyCTO during the process of data collection and were cleaned and checked for inconsistencies prior to analysis using STATA 13.0. Descriptive analysis, including means and proportions, were conducted for selected indicators of service quality to enable comparison between the two models. Qualitative data were coded manually using an Excel workbook containing preformed codes organized around primary and secondary research questions. Relevant excerpts from the KIIs and FGDs were systematically assigned to corresponding codes in the workbook. This coding process was followed by a thematic analysis using a deductive approach that triangulated information provided by various stakeholders and across stakeholder categories.

### Ethical considerations

Research Ethics Board (REB) approvals were obtained from PSI's REB and National Institute of Medical Research in Tanzania and written consent was granted by all qualitative and CEI participants prior to their participation.

## Results

### Institutionalization results

We observed that sociodemographic characteristics of adolescent girls who were reached under government-led implementation of Kuwa Mjanja were comparable to those reached by A360-led implementation (Table 2). The average age of adolescent girls reached through the program remained the same, but government-led events reached slightly more married, out-of-school adolescent girls with one or more children. Government-led events were also able to reach more adolescent girls in lower wealth quintiles than A360-led events.

**Table 2:** Client demographic and counseling outcome comparison between A360-led and government-led Kuwa Mjanja implementation.

Demographic/quality factor	A360-led (*n* = 413)	Government-led (*n* = 317)
Average age	17.2 years	17.5 years
Marital status	80% unmarried	70% unmarried
Parity	65% nulliparous	53% nulliparous
Educational status	40% in school	32% in school
Wealth quintiles	17% of rural girls are from bottom two wealth quintiles	25% of rural girls are from bottom two wealth quintiles
Method Information Index Plus (MII+)^a^	68% answered “yes” to all four questions	62% answered “yes” to all four questions
Quality of care	100% of girls reported being treated with respect by the provider	97% of girls reported being treated with respect by the provider
Prior use of contraception	24% of girls were using contraception prior to the event	27% of girls were using contraception prior to the event

A360, Adolescents 360

^a^ Method Information Index Plus (MII+) is a scale which can be used to measure informed choice. Its questions measure whether a woman received complete information about her options when she selected a contraceptive method. The MII + can also help predict whether a woman will continue their method. The MII + includes four questions: (1) Were you informed about other methods of contraception?; (2) Were you informed about possible side effects or problems you may have with the method?; (3) Were you told what to do if you experience any side effects or problems?; and (4) Were you told about the possibility of switching to another method if the method you selected was not suitable?.

**Figure 4:**
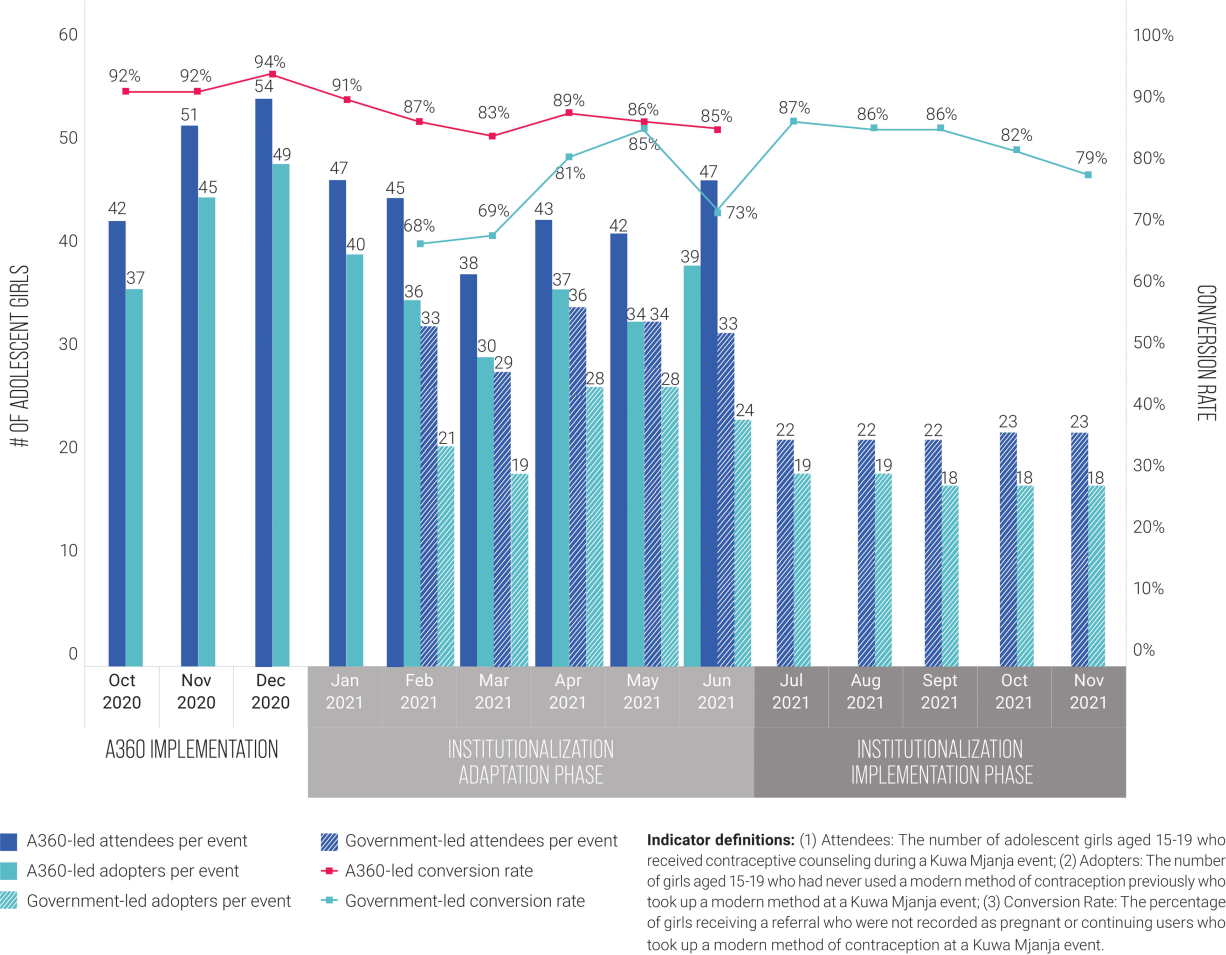
Girls reached, contraceptive adopters, and conversion rate per event, A360-led Kuwa Mjanja implementation compared to government-led implementation, October 2020 to November 2021.

The number of adolescent girls reached and adopting a modern method of contraception through each Kuwa Mjanja event declined under government-led implementation phase, with around 15 fewer girls reached on average and 17 fewer adopters per event (Figure 4). However, despite being lower than under A360-led implementation, government performance remained highly consistent over the course of the implementation period. Government-led events that took place during the adaptation phase had higher productivity than those that took place during the implementation phase, likely because A360 was still providing some direct support during the adaptation phase (for example, fielding co-mobilizers alongside government mobilizers), even when government was leading implementation. The percentage of event attendees who voluntarily took up a method of contraception (conversion rate) decreased slightly under government-led implementation, though remained similarly high at around 80%–85% over the course of the implementation period. In general, performance trends reflect a shift from an outreach-based model that has greater potential reach to one predominantly based in static facilities.

**Figure 5:**
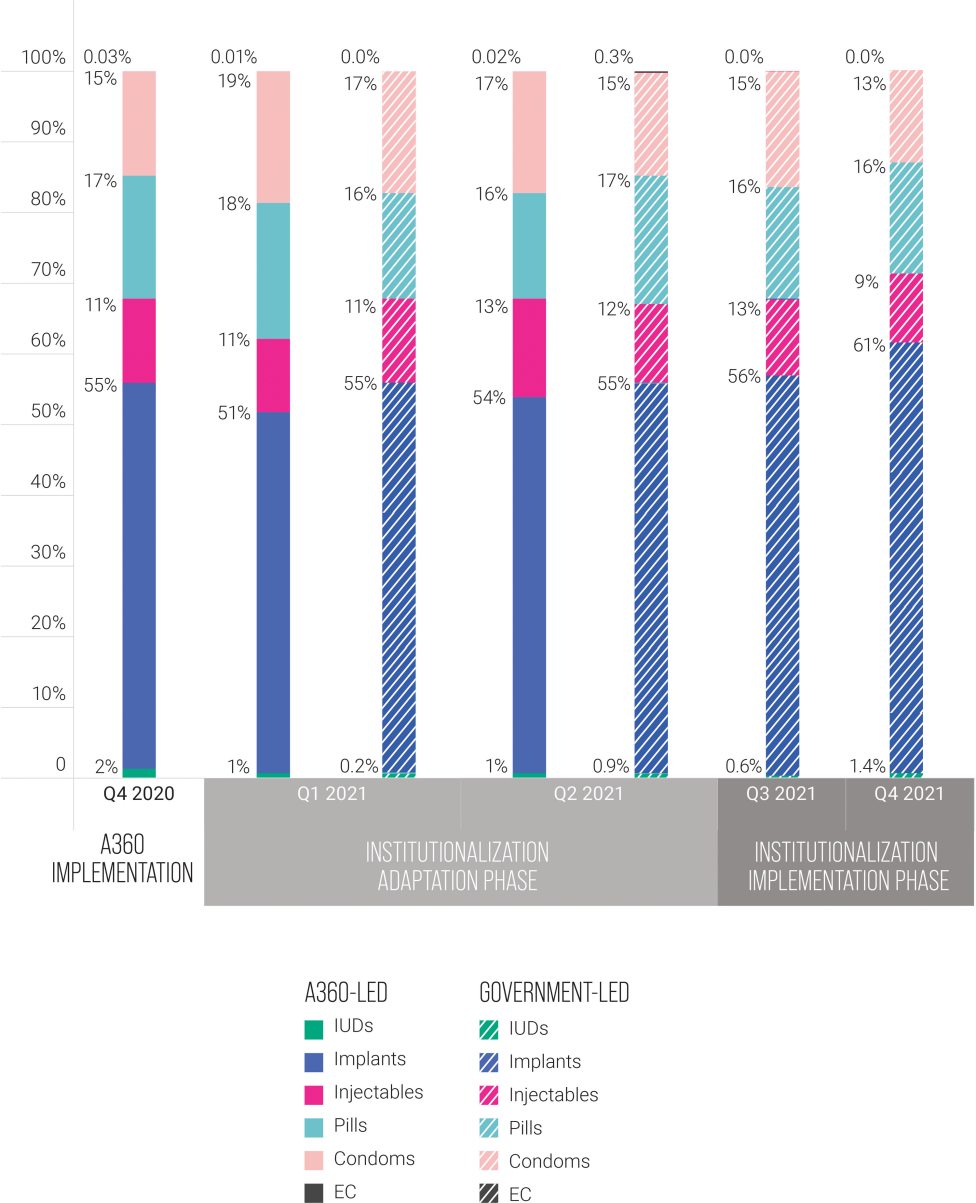
Adopter method mix by quarter, A360-led Kuwa Mjanja implementation compared to government-led implementation, Q4 2020 to Q4 2021.

The contraceptive method mix of adopters remained similar between A360-led implementation and government-led implementation (Figure 5). A slight increase was observed in the proportion of adopters who took up a LARC method. Most of this increase in LARC uptake was due to an increase in uptake of implants.

As detailed in Table 3, the key themes from FGDs and KIIs describing factors which enabled successful institutionalization of Kuwa Mjanja in government systems included (a) the commitment and collaboration of government stakeholders, particularly service providers and CHWs, and the presence of youth-supportive policies; (b) the alignment of the government-led model with the existing structures and systems and the establishment of school clubs through which girls were provided Kuwa Mjanja education; (c) prolonged engagement since the inception of A360 in Tanzania and capacity building efforts eliciting commitment of government stakeholders particularly service providers; (d) convergence between government authorities and nongovernment stakeholders in appreciation of adolescent pregnancy as a problem in the three regions; and (e) a user journey that delivered the anticipated client experience to adolescent girls even when adapted for government stewardship. As was expected, given the evidence that exists on effective institutionalization processes, model components that proved easiest to incorporate were those that had greatest existing alignment with government systems.

**Table 3:** Contextual findings on barriers and enablers to institutionalization of Kuwa Mjanja.

Key themes from qualitative research	Illustrative quotes
Enablers to institutionalization/institutionalization successes	
**Commitment and collaboration** Commitment and collaboration from government stakeholders, particularly service providers, was an important facilitator of Kuwa Mjanja institutionalization. Interest in seeing adolescent girls better supported motivated government stakeholders to adapt and iterate to create a Kuwa Mjanja model which could be operationalized within existing systems and resources.	“I would say commitment of the service providers themselves in the facilities is an important factor, you might go to a facility that has only two service providers, but they are committed to provide services, but go to another that has five providers but if there is no commitment it is a huge challenge. So commitment is the greatest pillar in ensuring these services are available.” (DRCHCo, Katavi) “… there was collaboration from the government right from the ministry level, the PO-RALG and I think they were supported very closely … down to the regional level, there was so much cooperation, that is why they could work in those regions and perform as much as they did.” (National MOH official)
**Existing alignment** SRH education components and contraceptive counseling and services had greater existing alignment with government systems and therefore proved easier to integrate. There were already existing systems in place to absorb these components, such as youth days at clinics and outreach activities in some schools. In the case of contraceptive counseling, this was something which was seen as appropriate for service providers to do and only required shifts to their approach to service delivery for adolescent clients.	“Providing SRH education and contraceptive counselling to girls as well as providing to them the methods that they choose has been possible and we can afford to provide these services in the absence of PSI.” (Service Provider, Tunduru, Ruvuma) “In the issue of providing education to the girls, we already had guidelines to do that and even partners have been using the government guidelines that were existent … it will not be a challenge and this part can easily be included in existing work plans because we have the guidelines and we have been using the same.” (RRCHCO, Rukwa)
**Extended engagement** Service provider capacity building and involvement of government stakeholders from the very beginning of A360's design process in 2016 and throughout implementation were successful at building government ownership. This included provision of youth-friendly services and LARC training for public providers, joint supportive supervision, and engagement of government stakeholders in routine program decision-making processes.	“… to me the involvement of the government was the most important factor, you can’t go to someone's house, don’t speak to them and then expect that you will work well with them. So involvement is very important and once someone is involved they must build ownership because they know exactly what is happening.” (RRCHCO, Rukwa)
**User journey fidelity** Overall, girls still experience the same broad “user journey” under government-led implementation as was provided under A360-led implementation, though maintaining the aspirational program components as well as follow-up for contraceptive continuation proved challenging.	“I really liked the event, the service provider taught us and made sure that we understood very well, the questions we asked were answered and this was done in a nice and friendly tone … we were also free to ask all the questions we had, like the side effects of the methods, the methods causing headache and things like that. They provided very friendly services; the provider acted like she was a friend.” (Adolescent Girl, Katavi) “… we felt free to access the services because they (event organizers) thought that if the event took place at school, then we would be afraid of our teachers. They sought permission for us at school and we went for the services. When at the health services, we sat at a place that was very far from the area where other daily services were being provided. The place where we received the education was outside, set apart for SRH education. When going to see the provider, we were shown the door that we were to enter, they made sure there was no one else in the room, they didn’t ask for our names or numbers.” (Adolescent Girl, Rukwa)
Barriers to institutionalization/institutionalization failures	
**Lack of budget and resource allocation** Lack of budget allocation was a key barrier to institutionalization, including compensation for mobilizers, incentives for service providers, supportive supervision, and quality audits. CCHP budgets could be leveraged to reserve resources for Kuwa Mjanja implementation, yet those resources were already stretched.	“We struggle a lot with CHWs because these do not have salaries or any formal payments, so they depend on services offered during outreach to get some amount of money. When we tell them there is no compensation for the mobilization activities, they become a little unwilling. We try to attach them to other activities that are compensated and ask them to also do KM mobilization for the same amount…. they agree to do it, but it will not be as efficient ….” (DRCHCo, Mpimbwe, Katavi) “The biggest barrier to institutionalization is the issue of budget, the government is supposed to set aside a budget so as to implement these activities. So even when we as PSI conduct mentorship and supervision, how will the providers and mobilizers be handled? If the budget was available, then it wouldn’t have been an issue to them.” (PSI Staff, Katavi)
**Misalignment with existing systems** Components which required resources outside the “norm” for the public health system, such as community outreach, vocational and entrepreneurship skills sessions, and payment for youth mobilizers, were more difficult to integrate. Skill sessions, for example, were clearly attractive to girls, yet the government did not have a source of funds to pay for the trainers and equipment needed to implement these components.	“The components that have been difficult for us to continue with … I would say outreach, because we only do the usual reproductive and child health outreach services which includes growth monitoring, FP services but we haven’t exactly focused on youth. Another thing is entrepreneurship, it has been difficult for us to integrate because of budget constraints.” (DRCHCo, Kalambo, Rukwa) “There has been a challenge with entrepreneurship component because there has to be a budget. The person that is to provide the entrepreneurship education needs to have materials, example he/she has to teach about making soap or tie-dye, there has to be materials so he/she can make these things. If there is no budget set aside for this, this person cannot provide this education.” (PSI Staff, Katavi)
**Lack of measurement prioritization** MNCH was more heavily emphasized in national and regional policy, procedure, and metrics than SRH. Regions where teen pregnancy rates were high had greater prioritization of ASRH, yet budget allocations were still determined by the national government based on regional performance against priority indicators identified at the national level. Most of these indicators are related to MNCH service delivery and outcomes.	“When implementing partners are phasing out, when the discussions of sustainability begin that is where the challenge is. For example, at the facility when you ask about the amount allocated for youths, YFS includes extra duty for SPs, mobilization/demand creation, etc., they would tell you that there is no specific allocation for youths in the budget. The government has other priorities like maternal deaths.” (PSI Staff, Rukwa)

DRCHCo, District Reproductive and Child Health Coordinator; YDO, Youth Development Officer; RRCHCo, Regional Reproductive and Child Health Coordinator; LARC, long-acting and reversible contraceptive; A360, Adolescents 360; CCHP, community council health plan; ASRH, adolescent sexual and reproductive health; SRH, sexual and reproductive health; PO-RALG, President's Office, Regional Administration and Local Government Tanzania.

The key themes describing the challenges to the institutionalization process were (a) inadequate resources and lack of budget, (b) core intervention components essential for Kuwa Mjanja's success that were misaligned with the public health system, and (c) the lack of prioritization within HMIS of measures of ASRH intervention success. From the study, it emerged that the lack of adequate numbers of youth-friendly providers who were trained to provide LARC remained a challenge within the overall Tanzanian health system. Furthermore, some Kuwa Mjanja components were core to program effectiveness but proved highly difficult to integrate. One such example was the aspirational or skills building program component. A360's aspirational program components describe an approach to contraceptive service delivery which begins with messaging around girls’ life goals instead of sexual activity and positions contraception as a tool for use to pursue their life goals. This component shifts messaging within mobilization and service delivery to begin with a validation of girls’ goals and establishing of contraceptive relevance. Though some government stakeholders took the “idea” of this program component and adapted it to fit their own constraints (organizing games/sports and soft skills training), overall institutionalization of this method was heavily constrained by available resources. Finally, it emerged that government stakeholders were more inclined to lay emphasis on tracking maternal, neonatal, and child health (MNCH) indicators such as skilled attendance at birth and maternal and perinatal morbidity and mortality than ASRH indicators. This prioritization impacted the way resources were allocated when developing annual plans and allocating budgets at national and regional levels. Since ASRH indicators were less prioritized, ASRH services were subsequently underfunded and holding frontline workers, district, and regional health personnel accountable for ASRH service delivery was constrained.

**Figure 6:**
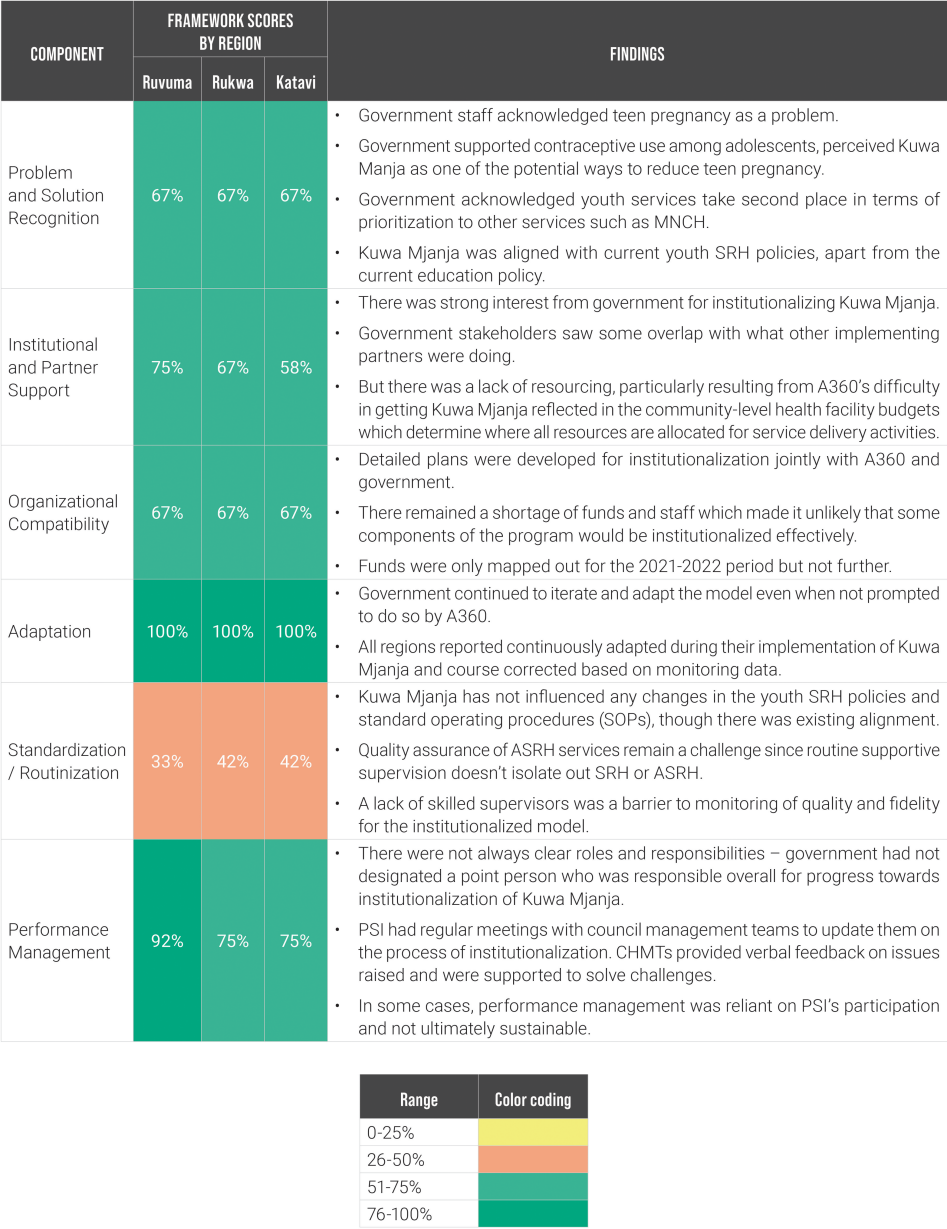
Milestone framework scores and contextual findings for Kuwa Mjanja institutionalization by priority region, Q4 2021.

A360 assessed each region using its institutionalization milestone framework on a quarterly basis during the first three quarters of 2021—this included collaborative scoring meetings with A360 and government stakeholders in attendance. In quarter four, A360 generated an external set of scores using qualitative research conducted by its learning partner (Figure 6). This external set of scores was developed using thematic analysis from interviews with government stakeholders.

## Discussion

The findings from this year-long accelerated government institutionalization process for Kuwa Mjanja provide clear learning to add to the global evidence base around barriers and enablers to effective institutionalization.

### Feasibility of institutionalization under narrow time constraints

Investing in the sustainable scale-up of adolescent-focused programming remains a critical component of countries’ pursuit of universal health coverage and the sustainable development goals (SDGs) (18, 19). Our learning during this institutionalization process for Kuwa Mjanja highlighted the significant potential of operationalizing user-centered ASRH models within existing government structures, even when working with a relatively narrow time frame. In A360's case, the convergence of its existing planning around institutionalization and sustainable scale and the decision by its funders to wind down implementation of Kuwa Mjanja in Tanzania prompted the project to explore what could be achieved over the course of a single year. In the process, we found that more could be achieved in that short period than we would have originally thought feasible. The demographic characteristics of girls reached as well as the quality of services delivered remained comparable under government-led implementation of Kuwa Mjanja even with the adaptations made to the model to make it operationalizable within existing government systems and resources. Adolescent girls also reported a similar experience with the institutionalized Kuwa Mjanja intervention. A360's routine involvement of government and implementation of Kuwa Mjanja through public structures during A360's first investment phase contributed to government's ability to take up the intervention's service delivery components effectively. Yet, Kuwa Mjanja was not originally designed for government institutionalization; therefore, this learning points to the potential for pursuing institutionalization even if projects have not begun with sustainable scale in mind. Although not trialed in the current study, a similar approach could be explored to establish the feasibility of embedding interventions such as Kuwa Mjanja into nongovernment health structures.

### Prioritizing “quick wins” compared to long-term impact

In keeping with consensus in the existing literature and knowledge about scale-up (3, 20), those components that were easiest to integrate had the greatest alignment with existing government systems. These “quick win” components—including the program service delivery components, client mobilization, and data reporting—provided “quick institutionalization success” for the project. Yet, there were other program components that were critical to program effectiveness but were much harder to integrate. Despite the tremendous results shown through government-led implementation, the difficulty in institutionalizing these core components—like the aspirational components of the program—provide a point of reflection for future projects who might be looking to pursue an institutionalization process as part of a project exit period with a focus on sustainability. The shorter time frame may necessitate paring down to even fewer, impactful components, which have the chance to make meaningful change. Programs like A360 must grapple with the tension between setting idealistic or pragmatic goals.

### Resource and measurement constraints as a key barrier to government institutionalization

Government stakeholders within A360's target districts showed tremendous commitment to operationalize a government-led Kuwa Mjanja model. However, even when commitment from individual stakeholders was high, a shortage of resources and competing priorities posed a severe challenge toward institutionalization of Kuwa Mjanja. This is a challenge echoed in the evidence base (14). What gets measured gets managed—local governments needed to meet their targets to receive funding and those indicators which absorbed most of their targets, in this case MNCH indicators, received highest priority. Shifting measurement to hold all levels of government accountable for ASRH service delivery is critically important, but this takes time and relies on heavy advocacy efforts. These advocacy efforts can not be dependent on one organization acting in isolation but must be a collective effort by multiple actors—including implementers and government. Additionally, even when these indicators are captured in HMIS, this does not guarantee prioritization if government policy and operational procedure does not explicitly mandate a focus on ASRH or if sufficient resources are not mobilized to support these activities. Institutionalization of evidence-based practices within key policy and operational documents can drive budget allocation. These institutionalization processes are similarly time-intensive but essential for long-term program sustainability. Despite the potential for some success in an accelerated institutionalization process demonstrated by A360's learning, this does not negate the necessity of pursuing earlier and longer-term institutionalization processes to generate maximum impact and program sustainability. Finally, we acknowledge the significant knowledge gaps in the area of institutionalization of promising or proven ASRH programs and this study provides a substantial contribution in closing this gap.

## Data Availability

The raw data supporting the conclusions of this article will be made available by the authors, without undue reservation.
